# Supplementary material for: Urbanization and flood risk analysis using geospatial techniques
Source: PLoS One. 2023 Oct 20;18(10):e0292290. doi: 10.1371/journal.pone.0292290 (PMC10588841; doi:10.1371/journal.pone.0292290)
Supplement: S1 File — (DOCX) [file pone.0292290.s001.docx]

**Description of supplementary material file**

**Change Matrices file**: This file contains data of Land use/land cover type structure of the study area, Land-use percentage change and rate of change during the two study periods as well as data in cross-tabulation format, that represents the change in Land use and land cover of the study area, which are computed from the classified LULC maps for two-time intervals, 1991-2002 and, 2002-2015. Tables 5 and 6 contain the transition matrix for each of the intervals; 1991-2002 and 2002-2015 respectively.
