# Supplementary material for: Urbanization and flood risk analysis using geospatial techniques
Source: PLoS One. 2023 Oct 20;18(10):e0292290. doi: 10.1371/journal.pone.0292290 (PMC10588841; doi:10.1371/journal.pone.0292290)
Supplement: Attachment — Submitted filename: Review report.pdf [file pone.0292290.s002.pdf]

Review Report

Manuscript Number: PONE-D-23-15951

Paper title: Urbanization And Flood Risk Analysis Using Geospatial Techniques

Journal: PLOS ONE

Overall comments

The research focuses on the analysis of urbanization and flood risk in the Greater Accra Region of Ghana. The study aims to map and analyze the trends of urbanization, identify flood risk zones, and analyze the relationship between urbanization patterns and flood disaster events from 1990 to 2015. The research also aims to contribute to the understanding of urbanization and flood disaster risk in the study area and similar regions. It emphasizes the importance of effective urban planning, land use policies, and regulations to minimize the expansion of urban areas and reduce flood disaster risk. I enjoyed reading the paper. However, I have some comments for further improvement of the paper. My specific comments are as follows:

Specific comments

Introduction:

The introduction provides a overview of the research objectives and the significance of studying the relationship between urbanization and flood disasters in the Greater Accra Region. It is important for this section to present literature review to be comprehensive, up-to-date, and critically analyze existing research on urbanization and flood risk. For example, it should describe how does urbanization contribute to increased flood disasters in Accra? What geospatial techniques were previously used in the analysis of flood risk? It should identify gaps in the literature and clearly articulate how the current study aims to address those gaps. However, it lacks specific details about the research gap and the novelty of the study. Additionally, it would benefit from a more explicit statement of the research questions or hypotheses.

Materials and Methods: The methodology section lacks specific details about the geospatial techniques used for mapping and analyzing urbanization and flood risk. While the study used ArcGIS Pro to analyze the data and identify areas of potential flood risk, it did not provide information on the accuracy of the results or any validation methods used to assess the accuracy of the flood risk mapping.

Results and Discussion:

The study did not provide a detailed analysis of the accuracy of the flood risk mapping results. While the study used data on topography, hydrology, land use, and historical flood events to map flood risk, it did not provide a detailed quantitative analysis of the relationship between urbanization and flood risk. This limitation may have resulted in an incomplete understanding of the extent to which urbanization contributes to flood risk in the study area. Could the authors address the issue?
